# Using live stream technology to conduct workplace observation assessment of trainee dental nurses: an evaluation of effectiveness and user experience

**DOI:** 10.1038/s41405-023-00132-0

**Published:** 2023-02-07

**Authors:** Caroline Taylor, Adalia Ikiroma, Anne Crowe, David H Felix, Gillian Grant, Lucy Mitchell, Teresa Ross, Margaret Saunderson, Linda Young

**Affiliations:** 1 Dental Care Professionals Workstream, NHS Education for Scotland, Edinburgh, UK; 2 Dental Clinical Effectiveness Workstream, NHS Education for Scotland, Edinburgh, UK; 3 Dental Directorate, NHS Education for Scotland, Edinburgh, UK

**Keywords:** Dental education, Health care

## Abstract

**Aim/objective:**

This study evaluates the effectiveness and users’ experience of using live stream technology to conduct workplace observation assessments of trainee dental nurses. Information on the usability, accessibility, and general satisfaction of this technological technique were collected.

**Materials and methods:**

This nationwide cross-sectional survey was conducted in Scotland and included one focus group and three online questionnaires with qualitative and quantitative questions. The quantitative responses were described using standard descriptive analysis, while the quantitative data were investigated using thematic analysis.

**Results:**

Eighty-one trainee dental nurses, 35 clinicians and 19 assessors participated in this study. Live stream observation was generally well received by the trainee dental nurses and clinicians, who thought that it had helped increase their confidence to perform practical skills. The assessors also stated that overall satisfaction was high, and that live stream observation met their expectations for efficacy. However, several technical challenges, such as network issues were brought up by responders.

**Conclusion:**

This study provides evidence that workplace observation assessments can be performed in the future by using live stream technology. However, additional investigation and comparison will aid in determining the most effective way of using this approach and providing feedback to promote learning among dental trainees.

## Introduction

The coronavirus disease-19 (COVID-19) pandemic resulted in global lockdown and created disruptions in educational delivery and assessment processes. Many educational activities including course completion examinations and certification were either postponed or cancelled. Of particular concern was the delivery of robust and timely observational assessment of practical skills [1]. Assessment is regarded as a crucial component in the learning process [2], and it should be sustainable if it is to foster lifelong learning [3]. This suggests that no matter what assessment method is utilised, it should effectively stimulate engagement to improve learning and performance, and thus promote sustainable development of learning capabilities. Emphasis on workplace‑based assessment (WBA) in dental health professionals’ training has been increasing in the last decade [4–7]. Most WBAs are observational and help the assessor to assess a trainee’s professional competence in a range of clinical scenarios. They also provide the opportunity for timely and objective feedback to the learner about both their clinical and non-clinical soft skills [7]. A key aim of feedback is to encourage and support learners to self-assess and reflect on their learning to help them self-correct and orient their current performance.

Dental Nurses are members of a group of Dental Care Professionals (DCPs) who play a vital role within a dental team and provide support and assistance in clinical and non-clinical aspects in providing high-quality patient care. All dental professionals must be registered with the General Dental Council (GDC) before they can work in the United Kingdom [8]. It is a requirement that dental nurses undertake a pre-registration training programme with a training provider approved by the GDC towards GDC recognised qualification. Mindful of this, Skills for Health recognised that assessors may find it more difficult to gain access to a dental environment to carry out observations during COVID-19 restrictions and authorised the use of live video stream as a suitable assessment method [9]. Skills for Health is a non-profit organisation committed to the development of an improved and sustainable healthcare workforce across the United Kingdom.

Observation of practice is a primary source of evidence and a fundamental principle of the Scottish Vocational Qualification (SVQ) in Dental Nursing qualification [10]. The SVQ standards require assessment methods to be selected on the basis of their internal verification process, which is made up of its validity, reliability, and practicability [11]. The validity of an assessment is the degree to which the assessment method is appropriate to the standards. The reliability of an assessment is the degree of consistent results when used with different candidates, different assessors and on different occasions. The practicability of an assessment refers to the feasibility that the assessment makes best use of available resources, equipment, and time. Prior to the COVID-19 pandemic, in order to conduct a WBA all assessors were required to travel to the trainee’s workplace. Depending on the geographic distance from the assessor’s base to the trainee’s workplace, conducting the observation might result in at least two visits which made a significant impact on travelling time and costs.

El-Kishawi et al. highlighted that evolving technologies and methods of assessment could enhance students’ learning environments and improve tutors’ assessment experiences [2]. Some studies have reported students’ positive learning experience from using digital technology for assessment in both medical and non-health disciplines [1, 12–16]. These studies have uncovered benefits at the tertiary education level such as increased access and flexibility [1], the provision of a platform for an interactive and independent learning opportunity [15], a useful tool for improving practice and assessing competency [12], and the opportunity to develop skills in the use of computer technology [13]. Additional benefits beyond skill acquisition were identified in a recent study which found that video feedback promoted greater knowledge retention, identified performance situation, clinical reasoning, self-efficacy, and learning satisfaction experience among students [17]. The outcome from video feedback was greater compared to computer assisted assessment analysis report or online-written comment.

To ensure uninterrupted progress and reduce the impact of the COVID-19 pandemic restrictions on trainee dental nurses, NHS Education for Scotland’s (NES) DCP Workstream devised the process to align the Skills For Health COVID-19 supplementary guidelines for the assessment strategy. With reports that the use of real-time online assessment has good prospects [1] and culminates in positive academic outcomes [18], a process utilising live video stream to conduct observational WBAs and effectively assess performance was designed and applied. Research exploring the use of digital technology has investigated the reliability and accuracy of live stream observation for performance assessment and feedback; [1, 19–23] however, research evaluating the use of live stream technology at workplace level is in its infancy. Wang et al. reported its usefulness and feasibility with high interrater reliability of video-based assessment of medical residents’ performance [21]. McQueen et al. highlighted the benefits of video-based assessment including the ability to capture clinical ability in the operating room without decreasing intraoperative efficiency and the potential to improve formative assessment and feedback practices [20]. Similarly, Rao et al. found that 85% of participants strongly agreed and 15% agreed that essential minor deviations during real-time online practical assessment did not compromise the basic principles and goals of assessment, and the quality of assessment through this mode of assessment was graded as good to excellent [1]. Therefore, the evidence suggests digital technology can transform the way workplace‑based assessment is delivered.

However, very little is known about the use of technology in performing workplace assessment in dental education. It is important to consider whether it can be objectively measured and evaluate users’ perceptions of its usefulness and effectiveness, particularly in the post-pandemic era. This knowledge is key to integrating and sustaining the use of live stream technology in vocational dental education programmes which are actively seeking opportunities to embed technology-based education to improve their programme. Therefore, the focus of this study was to explore the views of trainee dental nurses, clinicians, and assessors to evaluate users’ experiences and satisfaction of using live stream technology to conduct workplace observational assessments.

## Methods

### Design

This was a national cross-sectional survey conducted in Scotland comprising three online questionnaire and one focus group.

### Participants

The study participants included all trainee dental nurses undertaking pre-registration training with NES, the clinicians they provided chairside support to and assessors who completed the live stream observation assessment process. The clinicians were dentists, dental therapists, and hygienists who work with the trainees in their practice, and the assessors were Dental Tutors employed by NES, who carry out the role of an Assessor.

### Description of the Assessment Process

Evidence of candidates’ performance is drawn primarily from naturally occurring work activities that take place under normal working conditions in a normal work environment. This WBA requires an assessor to observe a trainee dental nurse while carrying out practical based tasks as defined in the qualification standards as they provide chairside support to a GDC registered dentist, hygienist, or therapist providing care to the patient. This is referred to as a Performance Criteria, and it describes the level of competency required for the SVQ in Dental Nursing qualification. This observation is conducted in the trainee’s own workplace, which could be a General Dental Practice, Public or Hospital Dental Service. Students pursuing the SVQ in Dental Nursing must be observed supporting the patient and the clinician as they provide chairside support during a variety of clinical dental procedures and duties within their role. The assessor records their performance and provides feedback on their findings. This provides evidence for the trainee’s portfolio to demonstrate achievement. Assessors must be competent in the area of practice to which the specific national occupational standards apply, according to the Scottish Qualifications Authority (SQA), and for the SVQ in Dental Nursing, assessors must hold a qualification recognised by the regulatory body for enrolment and maintain professional registration with the GDC as well as obtain an assessor qualification.

Microsoft Teams Platform (https://www.microsoft.com), a workspace for real-time collaboration and communication was used during the assessment. Microsoft Teams is freely available for download, with secure connection, and features that allow multiple participants and screen sharing. The meeting link was shared with all participants, allowing them to connect, the assessor to observe, and interact during the practical observation. Before the observation, a test run was conducted to ensure connectivity and video/audio quality.

### Questionnaire Development

There were three questionnaires developed for data collection: one for dental nurse trainees, one for clinicians, and one for assessors. The majority of the sections in the trainee and clinician questionnaires were the same. The trainee questionnaire was divided into five sections to collect data on (1) preparation (seven questions), (2) access and usability (four questions), (3) equipment (two questions), (4) support and feedback (four questions for clinicians and five questions for trainees), and (5) expectation [performance/overall satisfaction] (two questions). Both questionnaires included open-ended questions for comments on the aforementioned measures. The assessor questionnaire included 3-items to collect information about expectations: [effectiveness/overall satisfaction] (two questions) and an open-ended question about effectively assessing trainee performance.

Some of the closed-ended question items required a binary response (Yes/No). Most questions were based on a 3-point Likert scale with ‘3’ indicating never and ‘1’, all the time to statements in the access and usability domain; a 4-point Likert scale with ‘4’ indicating immediately and ‘1’, several days later to statements in the feedback domain; and a 5-point Likert scale with ‘5’ indicating strong agreement and ‘1’, strong disagreement to statements in the preparation, support and feedback, performance, effectiveness, and overall satisfaction domains, respectively.

### Data Collection

This was an online questionnaire survey hosted on Questback which is the NES approved online survey platform. This was distributed by email containing a secure link to the survey to the trainees and clinicians after completing the live stream observation assessment, whilst the assessors completed their questionnaires at the end of the observation assessment period. Data were collected from November 2020 for a period of two weeks to complete after the live stream observation assessment. In addition, invitations to participate in online focus groups via Microsoft Teams were circulated to all assessors employed by NES.

### Data Analysis

Standard descriptive analyses were performed to describe the responses using the Statistical Package for the Social Sciences (SPSS) version 27 [24]. For each, median (and interquartile range [IQR]) rating was used as central tendency (central quartile range) and internal consistency using Cronbach’s alpha or Spearman-Brown coefficient. A cut-off value of 0.6 was considered a good internal consistency of the items in the scale [25]. Items were reverse scored when necessary; therefore, the higher the score, the more positive the response. The open-ended data were explored using thematic analysis [26, 27] to identify themes which emerged from the data within the measures. This involved organising the qualitative data into themes and was performed on Microsoft Excel.

### Results

Out of 81 trainees that participated in the live stream observation, 45 completed the questionnaire with a response rate of 55.6%. Additionally,19 assessors participated in the live stream observation, although only 15 of them, together with 35 clinicians, completed the questionnaire. The response rate was 78.9% for assessors; however, it was not possible to calculate this for the clinicians as the total number involved in the trainee’s remote observation assessment is unknown. In line with the research question, the collected data provided information about the participants’ perspectives on the effectiveness and overall satisfaction of using live stream technology to conduct workplace observation competency assessment of trainee dental nurses. The internal consistency coefficient across all items for trainees, clinicians and assessors were 0.72, 0.82, and 0.81, respectively, Table 1.

**Table 1:** Median rating and reliability according to the measures for respondents.

Category	Measure^a^	x͂	IQR	α/*ρ*
Trainee				0.72
	Preparation	5.00	4.00–5.00	0.63
	Access and usability	3.00	2.00–3.00	0.63
	Feedback and support	5.00	4.00–5.00	0.61
	Expectation (performance and overall satisfaction)	4.50	4.00–5.00	0.69
Clinician				0.82
	Preparation	4.00	3.00–4.00	0.65
	Access and usability	3.00	2.00–3.00	0.80
	Feedback and support	3.00	3.00–4.00	0.68
	Expectation (performance and overall satisfaction)	4.00	3.50–4.50	0.84
Assessor	Expectation (effectiveness and overall satisfaction)	3.50	3.00–4.00	0.81

Note. x͂- Median; IQR- Inter-Quartile Range α/*ρ*-Cronbach’s alpha/Spearman-Brown coefficient

^a^The value for equipment domain was not included in the table as the internal consistency was not satisfactory

## Trainee and clinician questionnaire findings

### Preparation

Trainees agreed that they were prepared for the live stream observation (median = 5.0; IQR 4.0-5.0; α = 0.63), Table 1. The majority (93%) received the guidance document that provided information on the method of assessment and vital support before the observation. Ninety-six percent agreed that they followed the guidance for the observation, (93%) agreed that the guidance was discussed with their teams and (87%) agreed that their workplace provided support in preparation for the observation. Eighty-seven percent agreed there were able to test the process with an assessor before the observation and (89%) agreed that their workplace provided support in preparing them for the observation. In addition, (96%) of the respondents agreed that patients were comfortable with the method used for the observation, Table 2.

**Table 2:** Descriptive statistics for trainees and clinicians.

Measure/Questions	Trainees	Clinicians
Expectation (performance and overall satisfaction)		
*Allowed demonstration of performance as a dental nurse*		
Agree *n*(%)	45 (100)	29 (82.9)
Uncertain *n*(%)	–	5 (14.3)
Disagree *n*(%)	–	1 (2.9)
*Overall satisfaction of your experience*		
Excellent *n*(%)	20 (44.4)	8 (22.9)
Good *n*(%)	25 (55.6)	26 (74.3)
Poor *n*(%)	–	1(2.9)
Preparation		
*Received the live stream observation guidance document*		
Yes *n*(%)	42 (93.3)	32 (91.4)
No *n*(%)	3 (6.7)	3 (8.6)
*The instructions within the guidance was clear to follow*		
Agree *n*(%)	43 (95.6)	27 (77.1)
Uncertain *n*(%)	1 (2.2)	7 (20.0)
Disagree *n*(%)	1 (2.2)	1 (2.9)
*Discussed the content of the guidance document*		
Agree *n*(%)	42 (93.3)	29 (82.9)
Uncertain *n*(%)	2 (4.4)	5 (14.3)
Disagree *n*(%)	1 (2.2)	1 (2.9)
*Able to test the process before the observation*		
Agree *n*(%)	39 (86.7)	25 (71.4)
Uncertain *n*(%)	–	6 (17.1)
Disagree *n*(%)	6 (13.3)	4 (11.4)
*Workplace provided support in preparing for the observation*^a^		
Agree *n*(%)	40 (88.9)	32 (91.4)
Uncertain *n*(%)	4 (8.9)	–
Disagree *n*(%)	1 (2.2)	3 (8.6)
*Patients involved were comfortable with the method used*		
Agree *n*(%)	43 (95.6)	33 (94.3)
Uncertain *n*(%)	2 (4.4)	2 (5.7)
Disagree *n*(%)	–	–
*Felt comfortable using technology for the workplace observation*		
Agree *n*(%)	43 (95.6)	31 (88.6)
Uncertain *n*(%)	2 (4.4)	2 (5.7)
Disagree *n*(%)	–	2 (5.7)
Feedback and support		
*How soon was feedback received after the observation*		
Immediately *n*(%)	27 (60.0)	–
Same day *n*(%)	14 (31.1)	–
Next day *n*(%)	3 (6.7)	–
Several days later *n*(%)	1 (2.2)	–
*The feedback received: Timely / Shared the assessor feedback*^b^		
Agree *n*(%)	44 (97.8)	30 (85.7)
Uncertain *n*(%)	1 (2.2)	–
Disagree *n*(%)	–	5 (14.3)
*The feedback received: Was clear and constructive*		
Agree *n*(%)	45 (100)	29 (82.9)
Uncertain *n*(%)	–	6 (17.1)
Disagree *n*(%)	–	–
*The feedback received: Was helpful in supporting development*		
Agree *n*(%)	45 (100)	29 (82.9)
Uncertain *n*(%)	–	5 (14.3)
Disagree *n*(%)	–	1 (2.9)
*Provide any technology/IT assistance when needed*		
Yes *n*(%)	21 (46.7)	14 (40.0)
No *n*(%)	24 (53.3)	21 (60.0)
Access and usability		
*Difficulties accessing suitable equipment needed for the observation*		
All of the time *n*(%)	1 (2.2)	1 (2.9)
Some of the time *n*(%)	11 (24.4)	11 (31.4)
Never *n*(%)	33 (73.3)	23 (65.7)
*Able to access the live stream session with ease*		
All of the time *n*(%)	40 (88.9)	19 (54.3)
Some of the time *n*(%)	5 (11.1)	13 (37.1)
Never n(%)	–	3 (8.6)
*Connectivity reliable throughout the observation*		
All of the time *n*(%)	29 (64.4)	18 (51.4)
Some of the time *n*(%)	15 (33.3)	16 (45.7)
Never *n*(%)	1 (2.2)	1 (2.9)
*Able to achieve a clear view of the clinical area*		
All of the time *n*(%)	31 (68.9)	23 (65.7)
Some of the time *n*(%)	14 (31.1)	9 (25.7)
Never *n*(%)	–	3 (8.6)
Equipment		
*Type of IT equipment used*		
Android	3 (6.7)	1 (2.9)
Desktop/PC	2 (4.4)	4 (11.4)
Laptop	7 (15.6)	4 (11.4)
Tablet	5 (11.1)	3 (8.6)
Smart phone	28 (62.2)	23 (65.7)
*Additional equipment to hold/position device*		
Yes *n*(%)	17 (37.8)	12 (34.3)
No *n*(%)	28 (62.2)	23 (65.7)

^a^Different question for clinicians—Overall, did you feel that you received sufficient support from the assessor in preparing for the trainee’s observation?

^b^Trainee: The feedback received was: Timely; Clinician: Did the trainee share their assessor feedback with you?

Clinicians agreed that their trainees were prepared for the live stream observation (median = 4.0 IQR 3.0-4.0; α = 0.65), Table 1. The majority (91%) also received the guidance document that provided information on the method of assessment and vital support for the trainees before the observation. Over three-quarters (77%) agreed that the instruction within the guidance was clear to follow. Eighty-three percent agreed that they discussed the content of the guidance document with their trainee in advance of the observation. In addition, 71% agreed that they were able to test the process with the assessor before the observation and 94% agreed that patients involved in the live stream observation were comfortable with the method used. Eighty-seven percent agreed that they felt comfortable with using technology for the trainee’s observations. The majority (91%) of clinicians reported that overall, they felt that sufficient support was received from the assessor in preparing for the trainee’s observation, Table 2.

### Access and usability

Trainees agreed that the live stream observation was accessible and easy to use (median = 3.0; IQR 2.5–3.0; ρ = 0.63). Just under three quarters (73%) reported that they never had difficulties accessing suitable equipment to undertake the observation. Most (89%) reported that they were able to access the live stream with ease all the time to undertake the observation. In addition, more than half of the trainees (64%) reported that they had reliable connectivity all the time throughout the observation, and (69%) achieved clear view of the assessor to review their performance all the time, Table 2.

Clinicians agreed that the live stream observation was accessible and easy to use (median = 3.0; IQR 2.0–3.0; α = 0.80), Table 1. Around two-thirds of clinicians (66%) reported that they never had difficulties accessing suitable equipment to undertake the observation. More than half (54%) reported that they were able to access the live stream with ease all the time to undertake the observation. About half (51%) reported that they had reliable connectivity all the time throughout the observation, and around two-thirds (66%) achieved a clear view of the clinical area for the assessor to view their trainee’s performance all the time, Table 2.

### Equipment

For the equipment measure, the Spearman-Brown coefficient for both trainees (ρ = 0.36) and clinicians (ρ = 0.48) was <0.6, and therefore an overall measure was not calculated. For the type of IT equipment used for the observation, more than half of the trainees (62%) and around two-third (66%) of clinicians used smart phone while (16%) and (11%) used laptop. In addition, more than half (62%) of the trainees and around two-third (66%) of clinicians reported that they did not use additional equipment to hold or position their device to undertake the observation, while over a third 38% and just over a third (34%) used one to hold or position their device, Table 2.

### Feedback and support

Overall, trainees agreed that they were supported for the live stream observation (median = 5.0; IQR 4.0–5.0; α = 0.61), Table 1. Roughly half, 47% of respondents agreed that the assessor provided technology/IT assistance when needed, 98% agreed that the feedback from the assessor was timely, all respondents (100%) agreed that the feedback from the assessor was clear and constructive and supported their development, Table 2.

Overall, clinicians’ perspective towards feedback and support, was good, (median = 3.0; IQR 3.0–4.0; α = 0.68), Table 1. There was an equal agreement (83%) among clinicians with the statement “feedback received was clear and constructive” and “feedback received was helpful in supporting trainee development”. The vast majority (86%) of clinicians said the trainees shared their assessor feedback with them. However, when asked if the assessor provided technology/IT assistance when needed, more than half (60%) of clinicians said ‘no’, Table 2.

### Expectation

Overall, trainees agreed that using the live stream observation met their expectations (median = 4.5; IQR 4.0–5.0; ρ = 0.69), Table 1. To evaluate trainees’ expectation based on performance, all respondents (100%) agreed that the live stream observation allowed them to demonstrate their performance. For expectation based on overall satisfaction, nearly one-half (44%) reported their experience as ‘excellent’ and 56% had a ‘good’ experience from using the live stream observation for their assessment, Table 2.

Overall, clinicians agreed that using the live stream observation met their expectations (median = 4.0; IQR 3.5–4.5; ρ = 0.84), Table 1. To evaluate clinicians’ expectation based on performance, The majority (83%) of clinicians agreed that the live stream observation allowed their trainees to demonstrate their performance. For expectation based on overall satisfaction, just under three quarters (74%) of clinicians rated their experience as ‘good’ and over a fifth (23%) rated their experience as ‘excellent’, Table 2.

## Assessor questionnaire findings

### Expectation

In response to the statement probing the assessors’ expectations about the effectiveness of assessing the trainees using the live stream observation, overall, assessors agreed that using the live stream observation met their expectations (median = 3.5; IQR 3.0–4.0; ρ = 0.74), Table 1. Two-third (67%) agreed that they effectively assessed the performance of the trainees. However, a third (33%) were uncertain. For expectation based on overall satisfaction, the majority (87%) of assessors reported their experience as ‘good’, Table 3.

**Table 3:** Descriptive statistics for assessors.

Measure/Questions	Accessor
Expectation (effectiveness and overall satisfaction)	
*Enabled me to effectively assess the performance of trainee dental nurses*	
Agree *n*(%)	10 (66.7)
Uncertain *n*(%)	5 (33.3)
Disagree *n*(%)	–
*Overall satisfaction of your experience*	
Good *n*(%)	13 (86.7)
Poor *n*(%)	1 (6.7)
Undecided *n*(%)	1 (6.7)

## Qualitative findings from trainees’, clinicians’, and assessors’ questionnaires

Overall, 72 comments from trainees’, 49 comments from clinicians’ and 15 comments from assessors were obtained in response to the open-ended questions.

### Theme Identification

The respondents (assessor, clinician, and trainee) were asked to enter qualitative comments on relevant areas covered in the questionnaire. Responses for clinicians and trainees were coded into 5 domains (Preparation, Access and Usability, Equipment, Support and Feedback, and Expectation [Performance/Overall Satisfaction]) they were judged to best represent. Data corresponding to more than one domain were coded accordingly. Responses for assessors were related to effectively assessing using the live stream technology. Analysis of the data yielded distinct themes that represent the respondents’ experience of using the live stream technology.

## Clinician and Trainee

### Themes for Preparation

Both trainees and clinicians reported similar themes that enhanced their preparation for the live stream observation. Four distinct themes were identified that describe what it meant to participants to be prepared for the observation process. These four themes are *sense of responsibility, being organised, influence of time*, and *team collaboration*. The participants described how important these themes were with regards to the type of support provided by their team and their preparedness for the observation.

#### Sense of responsibility

The first theme, sense of responsibility was seen as imperative for respondents to use the live stream technology. Sense of responsibility means that the guidance document that provides the method of assessment and supports the vital preparation required for the observation was shared in advance. Here respondents expressed the significance of the guidance which they felt made a difference in the observation process.

One trainee reported, *“I was given instructions and guidance from my tutor prior to my observations in order to prepare. My tutor also set time aside prior to the observation to ensure set up was ok, which also helped to ensure there was no disruption on the day.”*

In addition, a clinician reported, *“I was guided by the trainees. They had done amazing well considering the situation. The girls have needed very little support as they have been committed and worked hard on their own initiative.”*

#### Being organised

For the second theme, being organised, participants indicated that this was also important in preparing for the observation. For example, the usefulness of trainees being well organised before and during the livestream observation is demonstrated by one clinician:

“*Our trainee managed and organised herself outstandingly on this aspect. To be perfectly honest it was all sorted without any input from myself. Obviously, my support was there should she need it. That goes for other members of the clinical team. Our trainee always explained to myself what her plans were and how she carried them out.”*

#### Influence of Time

Influence of Time as the third theme was expressed as experiences of dichotomous relevance of time where perceived support needs for the observation required an immediate response but once responded to, time became unimportant, with the respondents feeling well prepared for the observation. Trainees repeatedly expressed that they were supported with additional time to prepare for the observation.

One trainee expressed this as *“Sometimes it was hard to get observations within my workplace, but I got help around this when I needed to reach the deadlines*.” Another trainee also touched on the step taken to obtain more time for the observation process: *“Block the appointment for observations a bit longer than usual. It’s helps me enough time to setting up and finishing up.”*

#### Team collaboration

The last theme related to preparation, team collaboration, was expressed as support that trainees received from their team and the supporting clinicians, to prepare them for the live stream observation process. Trainees commented on the support from the administrative team who contacted patients and from colleagues who supported them by identifying ways to increase visibility during the observation. There was a general agreement for both trainees and clinicians that full support was needed to ensure that the trainees were prepared, knew what was happening and what was expected of them:

*“My employer purchased a tripod to hold my phone during the observations. Our reception team contacted the patient’s involved and explained the process.”* Another trainee responded that, *“We set up mock trials in each surgery to find out where would be best to have the phone sitting.”*

Clinicians expressed the importance of teamwork in providing support to the trainees:

*“We all work well as a team and feel the trainees were well supported.”* Also: *“Trainee had good support from head nurse, myself and from the associate they worked with. Between us we managed to find what would work best in terms of positioning, patient consent etc.”* This suggests that team support is a key element for ensuring effective preparation for and improved outcomes from the live stream observation.

### Themes for Access and usability

Three distinct themes were identified that informed what access and usability meant to the participants: *suitable alternative, technical constraints*, and *visual issues*.

#### Suitable alternative

Suitable alternative was a theme expressing that access and usability of the live stream technology worked well and live streaming is a flexible and significant tool that can be used to conduct workplace observation competency assessments. As one trainee said,

*“Great tool used to observe skills and it may be something to consider to continue using in foreseeable future because it is convenient and efficient.”*

Similarly, clinicians also reported how useful it was for the trainees to continue their clinical practice during the pandemic.

*“It was fine and enabled things to continue despite the current restrictions in clinical practice.”*

*“[Trainee’s] observation went [well] she knew what was expected of her and the technology worked fine.”*

One of the positive aspects of using the live stream technology was that whilst some respondents did report feeling anxious or stressed beforehand, when they began to use it, they reported feeling less anxious than they would have been in a face-to-face scenario:

*“I was very worried at the thought of the observation process when I started the course, but I think doing it via livestream actually made it less stressful and nerve wracking.”*

#### Technical constraints

Technical constraints were described as the main challenge encountered and identified as a barrier to access and usability of the life stream technology. Network issues, data usage and battery draining were regarded as problematic, especially as they were outside of the assessor’s control. However, some respondents highlighted their confidence in and positive experiences in using the live stream:

“*Had no problems at any stage, felt confident all elements were able to be observed and assessed properly*.” Another respondent emphasised that: “*I was nervous in case there was a drop in signal which would interrupt observation but found that all assessments took place well without too many difficulties*.” A third participant responded that: “*There were no issues with technology*.”

#### Visual issues

The last theme, visual issues, was expressed as not being able to have clear visibility of all aspects normally assessed within the observation process:

“*I’m not sure how reliable a static stream of working area allows for full observation and therefore full feedback in comparison to an assessor being present in the room*.”

However, some trainees highlighted how visibility issues were handled:

“*During one of the observations my assessor didn’t have a clear view and made me aware of this which was sorted accordingly with the help of the clinician and assessor*.”

### Themes for Equipment

One theme related to the need for additional equipment to hold and position the device for clearer visibility was identified: *portability*.

#### Portability

For the portability theme, most respondents indicated using a tripod as a platform for maintaining the stability of their device during the live stream observation process.

“*I used a large tripod which helped with the clear view of the surgery for my assessor*.”

However, respondents highlighted that other types of support, such as ring lights, phone cases, lab work trolleys and phone holders were used to support their devices during the process.

“*I have used a gooseneck phone holder. The phone holder is easy to secure on edge of table and it can be manipulated to what angle you want*.”

### Themes for Support and Feedback

Five themes informing support and feedback experiences included *process, visual support, educational value, technical support*, and *potential support if needed*.

#### Process

Process was expressed as clear communication support provided by the assessors during the live stream observation. It was expressed by one trainee as:

“*Because I was first student for observations, I didn’t get the document early enough before my observation, but I have been explained by my tutor verbally prior to my observation on the very same day*.”

Another clinician described this as an experience that left the trainee fully supported during the observation and seems to express this theme well.

“*Overall, our trainee has been given the highest standard of support. Where our trainee struggled, there was always an assessor there for guidance and support*.”

#### Visual support

The theme of visual support was expressed by respondents as experiences of knowing when the assessor does not have clear visibility. In this case, they can quickly inform the trainees about the visual support to enhance visibility. This theme encompassed several attributes including trainees’ and clinicians’ ability to know what to do when visibility is reduced. One trainee shared her experience of increasing visibility during the observation process:

“*During one of the observations my assessor didn’t have a clear view and made me aware of this which was sorted accordingly with the help of the clinician and assessor*.”

#### Educational value

Educational value was a third theme in support and feedback experiences and was described as experiences of interpersonal interactions during the observation process. These were characterised by perceptions of beneficial support, good, helpful, and useful information, and feedback towards the trainees and clinicians by the assessor. These experiences were felt to enhance the individual’s competency. This theme included attributes such as feeling comfortable and confident, flexibility of the assessor, being able to freely contact the assessor, and feeling good about the observation process because they were supported. One trainee highlighted how an assessors’ support led to a successful observation:

“*At the start of the observation the link wouldn’t allow me to join, my assessor was able to support me in giving me more help to access the livestream*.”

Educational value was seen as significant to support and feedback in the live stream observation process, as expressed in the words of one of the trainees: *“I enjoyed the live stream observation because the feedback I received made me feel confident in the fact that I was doing my job well and I will make a good dental nurse.”*

#### Technical support

Technical support as a theme represented difficulties experienced due to technological interferences of internet connectivity and troubleshooting technology support provided by the assessor. Respondents shared many experiences of how they were supported during technology problems and how personal examples of the assessor made a difference. One trainee shared,

“*I had problem with connecting my integrated camera and my tutor was telling me about her personal experience and how she sorted out that problem. I was very stressed about it and helped me out a lot to calm down*.”

#### Potential support if needed

The last theme, potential support if needed, was expressed as being able to receive technological support if required but it was not needed. One clinician stated,

“*I personally did not require technology support; however, our trainee was supported with any technical issues, and these were resolved*.”

### Themes for Expectation [Performance/Overall Satisfaction]

Two themes emerged for overall satisfaction with using the live stream technology: *negative* and *positive views for expectation*. Negative views mean respondents experienced some challenges when using the live stream technology and were not satisfied. Positive views, on the other hand mean that respondents experienced some valued benefit when using the live stream technology and were satisfied.

#### Negative views for overall expectation

Some negative views of using the live stream technology were identified. Respondents voiced concerns about technical problems such as network issues causing frequent disruption in the observation flow; system issues such as high-data usage and fast draining of battery; and infrastructural problems such as less visibility where assessors struggle to see clearly, and less value in using the live stream technology appeared in the response for overall satisfaction. Respondents thought that some of the challenges were compounded by the type of workplace building such as old buildings with thicker walls that restricted internet flow.

A trainee noted, *“… When connection wasn’t very good it became quite easy to get stressed to one ensure time keeping met while still trying to get good connection and also in case it got disconnected throughout.”*

Arranging specific patients for the observation due to busy clinical lists was seen as a problem by clinicians, and the live observation was thought to be less useful.

A clinician shared, *“Due to Covid, live stream was the only way of getting some form of observation, though there’s not much the assessor can adequately see to assess from a single, fixed vantage point. It was difficult to try to arrange specific patients in a row for the observations due to having a busy clinical list…I’m not convinced the live observation was useful in our nurse’s training.”*

#### Positive views for overall expectation

Some positive views of using the live stream technology included ease of using the live stream technology; how less stressful it was in comparison to face-to-face assessment; how well trainees coped; the ability to complete the observation on time; how confident trainees felt in being observed virtually; ease of contacting the assessor; and clear visibility. The predominant positive view reported by both trainees and clinicians was how the live stream technology worked well to ensure the assessment of trainees was performed during the pandemic.

One trainee shared: *“It was good overall as was quite flexible.”*

Another trainee highlighted, “*Live Streaming observation was positive in the way that due to the current circumstances I was still able to be observed in my work, there was a couple of times due to working in a hospital setting it required slightly more planning but overall, I was happy I was able to continue with my course and do my observations due to the circumstances.”*

It was recommended that recording and compiling individual treatment would be much easier. Nevertheless, despite these concerns other respondents also expressed that the use of the live stream technology was an appropriate alternative. This is shown in some clinicians’ responses:

“*Overall fantastic”. “I think the process of live stream assessment generally worked well in this scenario*.”

## Assessor perception for effectively assessing performance using the live stream technology

To effectively assess performance using live stream technology, the factors which shaped the assessors’ perceptions have been investigated. In this case assessors were asked to comment on whether the live stream observations enabled them to effectively assess the performance of trainee dental nurses. Assessors’ perceptions of the effectiveness of using the live stream technology varied, and mixed responses were received. The findings have been grouped into two categories: factors aiding effectiveness and factors inhibiting effectiveness. Factors aiding effectiveness were *efficiency, self-efficacy, clear visibility, cost-effectiveness*, and *reduced travel time*. Factors inhibiting effectiveness were *technical glitches* (Wi-Fi connection, high data usage and sound quality), *limited visibility, first time use of digital observation*, and *preferred method of observation* (face-to-face/blended approach).

Most assessors highlighted ‘some sort of [effective] strength’ associated with using the live stream technology. One assessor said, “*Most candidates coped well, and you were able to see enough to note.”* Another assessor expressed that he or she: *“Was able to carry out effective observation of candidates performance during a range of clinical dental procedures.”*

The method used for the observation was felt to be all important. Another assessor emphasised on the tone of cost saving that: *“There are a lot of benefits no travel time or costs, if patient cancels no time wasted for assessor, candidate can set up an observation last minute with assessor.”* Being a suitable alternative was also viewed as equally significant: *“Live stream observations are certainly an obvious alternative to in-practice observations.”* In addition, *“Live stream observations have worked well. Initially there was some reluctance from practitioners and there has been slight issue with sound at times but apart from that all good.”*

However, several assessors questioned the effectiveness of using the live stream technology, discussing factors that inhibited its use. The difficulty of measuring effectiveness was raised with concerns of technical glitches, most assessors voiced concerns regarding WIFI connection, data usage, sound quality, and limited visibility. One assessor highlighted, *“For some yes, but others no, very much depended on view and WIFI connection.”* Nevertheless, while some felt that there were some inhibiting factors of effectively assessing the trainees, others reserved criticism on the basis of unprecedented circumstances that could be resolved.

“*It can be difficult to see everything and is connection dependant, but I set up the meeting before the appointment time so that I can observe the candidate setting up for the procedure and I ask them to pan round the surgery. This also gives me the opportunity to ask any questions therefore fewer questions at the end of the observation if the student does not have much time*.”

Another assessor expressed, *“…What I was not able to capture, I questioned my student after the procedure or arranged for a TEAMS meeting when suitable for my candidate. Only once did I re-observe the procedure.”*

Among factors related to the effectiveness of using the live stream technology, a preference for in person observation was a view held by some assessors, *“I feel nothing can beat a face-to-face observation, meeting with the student, the staff and looking at the environment and building relations.”*

Another assessor emphasised, *“It is very much depending on the student, ability to get a good view of the working area and how much you can actually hear, especially regarding communication with both the patient and the dentist. Still feel a blended approach is a must to fill in the blanks/make confident assessment decisions.”*

## Focus group findings from assessors

We conducted three focus groups (*n* = 4, 4 and 5 participants, respectively) exploring assessors’ thoughts and experiences of using the live stream observation with the following aims: first, to identify potential information not already identified in the survey; and second, to derive a meaningful assessment of user experience in live stream observation as a group. The analysis of the focus groups identified three core areas of reflections relevant to using the live stream technology for workplace assessment: *Benefits* (Convenience such as ability to organise observations in limited time, ease of giving feedback, flexibility an opportunity to build relationship with missed dental practices; Time effectiveness and cost effectiveness such as increased time for trainees to ask questions, reduced travel time and possibility of short notice observation; reduced pressure); *Challenges* (Technical difficulties such as poor internet connections, sound quality and less visibility; losing skills observed during face-to-face observations); *Achievements* (Ability to support trainees with better visibility during the observation process e.g., using phone holders that can hang to the wall; ability to complete qualifications during a pandemic and upskilling of assessors by embracing this new environment to conduct the workplace assessment).

## Discussion

This study contributes to knowledge in dental education as it reports on an innovative strategy that has the potential to change the way in which workplace assessment is conducted. Consistent with previous research into digital technologies for assessment e.g. [1, 13–15, 22], we found that the benefits of using live stream technology for conducting the workplace observation assessment significantly outweighed the challenges encountered. This use of digital technology strategy seems to enhance sustainable teaching, learning and assessment particularly during the COVID-19 pandemic. The implementation of this innovative method has been successful and enabled assessment and progression of pre-registration dental nurses throughout the COVID-19 pandemic. The successful implementation required assessors who created a consistent approach and supported the trainees to be familiar with the process. In addition, removing the requirement for an assessor to travel to visit a trainee’s workplace to conduct the observation assessment and overall, significantly reduced time taken to undertake this assessment process and cost of travel. Overall providing a more flexible, sustainable approach to arranging this method of assessment. To the best of our knowledge, this is the first study to evaluate the experiences of trainees’, clinicians, and assessors’ together on the use of live stream observation.

The reliability of this study lay at an average of alpha value greater than 0.6 across the instruments which is acceptable and there was a good response rate to completing the questionnaires. The study represents a major change in workplace observation assessment. The live stream observation was highly beneficial for assessors as it decreased the need for in-practice assessment and efficiently compensated for the disruption of the trainees’ assessment. Overall, most trainees viewed the live stream observation process positively, including preparing for the observation process, access and usability, feedback and support, and satisfaction. This is consistent with the findings from a study assessing undergraduate nursing students’ perspectives towards video assessment of clinical skills that reported that students accepted the video assessment process positively [14]. The expectation component based on performance is an important strength of using the live stream technology.

All the trainees indicated that the live stream observation enabled them to demonstrate their performance as dental nurses. The trainees were expected to demonstrate their capability for safe and effective practice and maintain sufficient flexibility for providing patient care while using live streaming. Based on our findings, the live stream observation offered a safe working space, provided opportunity for the trainees to progress with their training, receive support from their assessors and feel confident about their job. In agreement, most clinicians (82.9%) indicated that the live stream observation enabled their trainees to demonstrate their performance as a dental nurse. This contrasts with the report of [15] who highlighted some concerns such as nerves and performance being affected by using video assessment but indicated its usefulness for assessing competency with regards to feedback. However, our findings align with Kam and others (2019) who reported increased performance competence of core basic nursing skill among participants that received video feedback [17]. In this study, the trainees also shared how they had learnt from the personal experience of the assessors to remain confident during the assessment process.

The live stream observation therefore allowed the trainees to present to the assessors how their practical experiences at the practice had culminated into new insights, technological experiences, and new learning. In addition, all the trainees reported their overall satisfaction experience with using the live stream was good or excellent. Consequently, clinicians also expressed high levels of overall satisfaction with using the live stream technology. Furthermore, (86.7%) of assessors reported that they had a good or very good overall satisfaction experience with the live stream observation. This is a significant transformative outcome for technology-based workplace assessment in dentistry. Guze (2015) highlighted the transformative learning experiences associated with using technologies that engages the learner and allows learning that does not endanger the patient [28]. It is also expected that assessors should be able to support learning into a more collaborative, personalised, and empowering experience [28].

Across the three groups, median overall satisfaction rates were higher for trainees and clinicians than assessors (4.0, 4.0 and 3.0, respectively), which represents good to very good experience among respondents. In addition, the median effectiveness score for assessors was 4.0, which shows that the assessors agreed with effectively assessing the performance of the trainees using the live stream technology. These results suggest that trainees, clinicians, and assessors had a positive experience of using live stream observation for the workplace observation assessment, and its use had an effective impact on trainee performance outcome and achievement. Given the changing situation, as this method of assessment is increasingly used, the assessors will likely better understand its benefit to improving trainees’ performance outcomes and reflective learning and their satisfaction rate will reflect supporting learning effectiveness.

However, a key challenge in using live streaming is the technology issue around internet stability which a concern for the trainees, clinicians, and assessors, as some were disconnected during the observation process. This may affect their abilities to be coordinated when performing the task during the observation process. Similarly, using live streaming can constitute physical barriers for trainees who may be perplexed by their requirements, structure, and navigation. Nevertheless, to combat this technology issues, before the observation, a guidance document on this method of assessment and support for vital preparation was sent to the trainees and during the assessment process, the assessors supported the trainees with a variety of troubleshooting techniques. The trainees, however, were positive about using live stream observation as a tool for their workplace assessment stating that the guidance document provided helped them prepare to use method for the assessment, allowed them to arrange a suitable time with their assessor and worked in a less stressful and nerve-wracking environment, even when visibility was reduced. This is because the assessors made them aware they didn’t have a clear view and advised on best position to enhance visibility, which made the observation process run smoothly.

## Study limitations and strength

The limitation of the study is centred around the limited published data to support the use of live stream observation as a method of workplace-based assessment. As this study involves a small number of dental nurse trainees, the findings may not be a reflective of the personal experiences and viewpoints of all dental nurse trainees, clinicians, and assessors of other training providers. However, a key strength of the study is that it investigated views and experiences of using live stream observation from different perspectives and took a mixed methods approach to validate the findings. Moreover, this research indicates that this method was effective, and support from NES’s DCP workstream team significantly contributed to the successful implementation. It is hoped that this paper can support assessors in dentistry and other disciplines in the implementation of using technology to conduct workplace assessment. The findings from this study and further analysis will inform where continual improvements can be made to effectively support students and workplace teams in the organisation, and facilitation of remote workplace observation competency-based assessment via live video stream.

## Conclusions, recommendations, and future work

Our main contribution in this paper is to investigate the views and experiences of using live streaming for workplace observation assessment to assess the overall satisfaction and effectiveness. Overall, it was found that satisfaction of using live streaming can be linked with the resolving technology issues and support received, but effectiveness is mixed. Our results, as well as the limitations of supporting data, point naturally to several directions for future work. However, sustaining this method and the future use of remote live observation will be dependent on the Skills for Health decision to maintain this as an assessment method with the assessment strategy for the SVQ in Dental Nursing qualification. Nevertheless, the use of remote live observation assessed a range of generic and practical trainee competencies that require a higher level of confidence in managing the technology used and carrying on with the assessment. It allowed for feedback and validation of the trainee learning experiences and was viewed as an efficient and effective way of assessing and providing feedback particularly in response to global pandemics such as the changing condition posed by the ongoing COVID-19 pandemic. Thus, it is a feasible option for assessing trainees based in areas with transport difficulty (for example, practices in the Western Isles) without having to travel.

NES has created a vision for Technology Enhanced Learning that will enable education by facilitating appropriate, high-quality Technology-Enhanced Learning for the Health and Social Care workforce now and in the future. The findings and learning from this project will be shared to support the integration and staff development of utilising the functionalities of various technologies within blended learning programmes as a robust assessment tool. Therefore, as the use of remote live observation continues to be developed, consideration should be given to incorporate features that enable acceptance and re-imbursement for high-speed internet/phone expenses. Further local research and comparisons will help to identify practical and efficient ways of using live streaming to conduct workplace-based assessment and give feedback to encourage learning among dental trainees. Nevertheless, the use of the live stream technology generally worked well for the purpose of the workplace-based competency assessment. What was learned from the experience with the COVID-19 pandemic is the need for flexibility concerning conducting the workplace-based assessment.
